# Identification and validation of a novel 16-gene prognostic signature for patients with breast cancer

**DOI:** 10.1038/s41598-022-16575-8

**Published:** 2022-07-19

**Authors:** Zhenhua Zhong, Wenqiang Jiang, Jing Zhang, Zhanwen Li, Fengfeng Fan

**Affiliations:** Department of Breast Center, Ningbo Women and Children’s Hospital, No. 339 Liuting Street, Ningbo, 315012 Zhejiang China

**Keywords:** Cancer, Computational biology and bioinformatics, Oncology, Breast cancer, Cancer genetics

## Abstract

Despite increased early diagnosis and improved treatment in breast cancer (BRCA) patients, prognosis prediction is still a challenging task due to the disease heterogeneity. This study was to identify a novel gene signature that can accurately evaluate BRCA patient survival. The gene expression and clinical data of BRCA patients were collected from The Cancer Genome Atlas (TCGA) and the Molecular Taxonomy of BRCA International Consortium (METABRIC) databases. Genes associated with prognosis were determined by Kaplan–Meier survival analysis and multivariate Cox regression analysis. A prognostic 16-gene score was established with linear combination of 16 genes. The prognostic value of the signature was validated in the METABRIC and GSE202203 datasets. Gene expression analysis was performed to investigate the diagnostic values of 16 genes. The 16-gene score was associated with shortened overall survival in BRCA patients independently of clinicopathological characteristics. The signalling pathways of cell cycle, oocyte meiosis, RNA degradation, progesterone mediated oocyte maturation and DNA replication were the top five most enriched pathways in the high 16-gene score group. The 16-gene nomogram incorporating the survival‐related clinical factors showed improved prediction accuracies for 1-year, 3-year and 5‐year survival (area under curve [AUC] = 0.91, 0.79 and 0.77 respectively). *MORN3, IGJ, DERL1* exhibited high accuracy in differentiating BRCA tissues from normal breast tissues (AUC > 0.80 for all cases). The 16-gene profile provides novel insights into the identification of BRCA with a high risk of death, which eventually guides treatment decision making.

## Introduction

BRCA (BRCA) is the most prevalent female malignancy in US and China. An estimated 284,200 cases will be diagnosed and 44,130 patients will die of the disease in 2021, accounting for more than 15% of newly diagnosed cancer cases and 7.3% of cancer-related mortalities^1,2^. According to the molecular classifications, BRCA can be mainly divided into five subtypes: luminal A, luminal B/human epidermal growth factor receptor 2[Her2] negative, triple positive (ER+, Progesterone receptor [PR]+, Her2+), Her2-enriched, and triple negative (ER−, PR−, Her2−)^3^. With the significant progresses of medical technology, the prognosis of BRCA has been remarkably ameliorated. However, the prognosis is still not optimistic for BRCA patients diagnosed at late stages.

The development of methods for risk stratification in BRCA has been a hotspot of research. Several studies demonstrate that multigene signatures might be more accurate for risk stratification than the traditional approaches in BRCA^4,5^. The MammaPrint, a 70-gene signature, is a prognostic model to stratify node-negative BRCA patients with different survival probabilities^4^. Oncotype DX is a 21-gene signature that provides information of the likelihood of recurrence and weighs the potential benefits of chemotherapy in the node-negative, estrogen receptor positive BRCA^5^. These multigene assays show potential clinical utility, but still need to be validated in large, randomized trials^6^. Moreover, the established methods are applicable to only limited disease subtypes, there is still lack of an effective prognostic model that could be used for almost all BRCA subtypes.

In the current study, we aimed to develop a novel gene profile to accurately estimate disease prognosis. We first examined all genes for their association with overall survival (OS) using the gene expression and clinical data of The Cancer Genome Atlas (TCGA) dataset^7^ and validated the results in the Molecular Taxonomy of BRCA International Consortium (METABRIC)^8^ and GSE202203^9^ datasets. We next established a 16-gene score based on a linear combination of 16 gene expression levels and 16-gene nomogram to precisely predict the overall survival (OS) of BRCA patients. Lastly, we performed expression analysis of 16 genes and demonstrated their diagnostic values in BRCA.

## Methods and materials

### Data acquisition and processing

We obtained RNA-seq expression data and clinical data of BRCA patients from the two different sources, the first of which was the TCGA database (n = 1080 patients), the second source was the METABRIC study which was used to validate the associations between gene expression and OS (n = 1904 patients). Clinical features of BRCA patients are summarized and presented in Table1 respectively. As the gene expression unit of the TCGA dataset differs from that of the METABRIC cohort, normalization of gene expression was performed using the formula z = (x-$\overline{x }$)/s. where x, $\overline{x }$ and s are the gene expression value, mean and standard deviation of gene expression values. This study was in compliance with strict confidentiality guidelines and regulations regarding personal data protection, all methods were carried out in accordance with relevant guidelines and regulations. We also obtained 2913 breast cancer patients from the GEO database (GSE202203) to validate the association between gene expression, risk score and overall survival^9^.

**Table 1:** Association between the clinical features and breast cancer patients’ mortality in the TCGA and METABRIC datasets.

Variables	The TCGA dataset			The METABRIC dataset		
	Alive	Dead	P value	Alive	Dead	P value
Age	57.95	61.22	0.01^a^	56.46	64.44	< 0.001^a^
Tumor weight	367.54	395.57	0.5^a^	23.32	28.36	< 0.001^a^
Number of positive lymph nodes	2.07	4.3	< 0.001^a^	1.21	2.57	< 0.001^a^
**Menopausal stage**			< 0.001^b^			< 0.001^b^
Indeterminate	19	15				
Peri-menopause	38	1				
Post-menopause	602	91		562	931	
Pre-menopause	209	18		239	172	
**ER status**			0.07^b^			0.41^b^
Positive	694	100		606	853	
Negative	196	41		195	250	
**HER2 status**			0.15^b^			0.16^b^
Positive	137	23		89	147	
Negative	499	57		712	956	
**PR status**			0.1^b^			0.33^b^
Positive	600	86		435	574	
Negative	286	56		366	529	
**Cancer stage**			< 0.001^b^			< 0.001^b^
I	163	16		263	216	
II	549	66		318	482	
III	202	46		29	86	
IV	4	15		1	8	
**T stage**			< 0.001^b^			
T1	241	33				
T2	550	77				
T3	112	25				
T4	24	15				
**N stage**						
N0	467	44	< 0.001			
N1	296	59				
N2	96	22				
N3	61	15				
**M stage**						
M0	772	120	< 0.001			
M1	10	17				
**Chemotherapy**						0.05^b^
Yes	450	36	0.89^b^	184	212	
No	257	22		617	891	
**Hormone therapy**						0.2^b^
Yes	247	17	0.47^b^	480	694	
No	460	41		321	409	
**Radiotherapy**						< 0.001^b^
Yes	374	58	0.05^b^	530	607	
No	497	51		271	496	

a and b indicate student t test and fisher exact test respectively.

### Identification of survival-related clinical features and genes

We aimed to identify survival-related clinical features using different statistical methods. For quantitative variables, we utilized student t test to characterize their associations with OS. With respects to qualitative variables, we implemented fisher exact test to investigate their associations with OS. We followed Sha et al.’s methods^10^ to identify and classify survival-related genes. In brief, we firstly split BRCA samples into two subgroups, the low-expression and high-expression groups, based on the median expression value. We performed Kaplan–Meier survival analysis to evaluate the statistical significance of the differences in OS with the survival package^11,12^ and conducted multivariate Cox regression model to further validate the survival analysis. Survival-related genes with odd ratio [OR] > 1were considered as risk genes, while genes with 0 < OR <  = 1 were defined as protective genes. To further evaluate the prognostic importance of the 16-gene score, we drew receiver operating characteristic (ROC) curves and computed the area under curve (AUC) values using the R package pROC^13^. To investigate the potential biological function of prognosis-related genes, we analyzed the enrichment of Gene Ontology (GO) term and Kyoto Encyclopedia of Genes and Genomes (KEGG) signalling pathway using the online tool g:profiler^14^.

### Establishment and validation of the 16-gene score

We followed Lai et al.’s methods^15^ to choose the set of genes which performed best in prognosis prediction and develop the 16-gene risk score. In brief, the least absolute shrinkage and selection operator (LASSO) models comprising different number of genes were evaluated for prediction accuracies of OS using glmnet in the TCGA dataset^16^. The 16-gene score was created using the following formula: 16-gene score = − 1.91 + expression of gene 1 × β1 + expression of gene 2 × β2 + ⋯ + expression of gene n × βn. β values represented the coefficients generated from the optimal LASSO model. We then implemented Kaplan–Meier survival analysis, multivariate Cox regression analysis and stratification analysis to further investigate the association between the 16-gene score and OS in BRCA. We also analyzed the prediction capability of the 16-gene score for progression-free survival (PFS) and disease-free survival (DFS) in the TCGA cohort using Kaplan–Meier survival analysis. Lastly, we utilized linear regression model to investigate the correlations between clinical characteristics and the 16-gene score in the TCGA and METABRIC cohorts. P < 0.05 was considered statistically significant.

### Gene set enrichment analysis

On the basis of the median 16-gene score, the BRCA patients were split into two subgroups: the high and low 16-gene score groups. Gene set enrichment analysis (GSEA)^17^ was implemented to determine the dysregulated signalling pathways related to the 16-gene score using the default parameters. Q value < 0.25 was considered statistically significant.

### Construction and validation of the 16-gene nomogram

Nomogram was constructed using the rms package in R, and included patient’s age, tumor stage, menopause status, number of positive lymph nodes and 16-gene signature as they are significantly correlated with OS of BRCA. The performance of the nomogram developed was evaluated in the TCGA cohort and validated in the METABRIC cohort using the R package pROC. AUC values were computed accordingly for the nomogram in the prediction of 1-year, 3-year and 5-year survival.

### Expression analysis of prognosis-related genes

The online server cbioportal^18^ was utilized to analyze the mutational profiles of the 16 genes in the TCGA cohort. Furthermore, the expression data of 779 BRCA tissues and 100 paired non-cancerous tissues were downloaded from the TCGA database. Differentially expressed genes were determined between BRCA tissues and paired normal tissues using student t test. To investigate the diagnostic values of the 16 genes, the pROC package was used to determine whether the gene expression could effectively distinguish cancer tissues from paired normal ones. P value was adjusted using false discovery rate. Adjusted P < 0.05 indicated statistical significance.

### Ethical statement

As all the data were obtained from public databases, the study didn’t need to be approved by the Ethics committee of Ningbo Women and Children’s Hospital.

### Consent to participate

Informed consent was obtained from all individual participants included in the study.

### Consent to publish

The authors affirm that human research participants provided informed consent for publication of this study.

## Results

### Identification of survival-related clinical features in BRCA

We initially performed survival analysis between clinical features and OS and revealed higher patient’s age, more positive lymph nodes, higher cancer stage, clinical T stage, clinical N stage, clinical M stage, post-menopause were high risk prognosticators for OS in the TCGA cohort (P < 0.05 for all cases, Table1). Furthermore, the inverse correlations between overall survival and patient’s age, more positive lymph nodes, higher cancer stage, post-menopause, tumor size, radiotherapy were independently validated in the METABRIC cohort (P < 0.05 for all cases, Table1).

### Identification and validation of survival-related genes in BRCA

**Figure 1:**
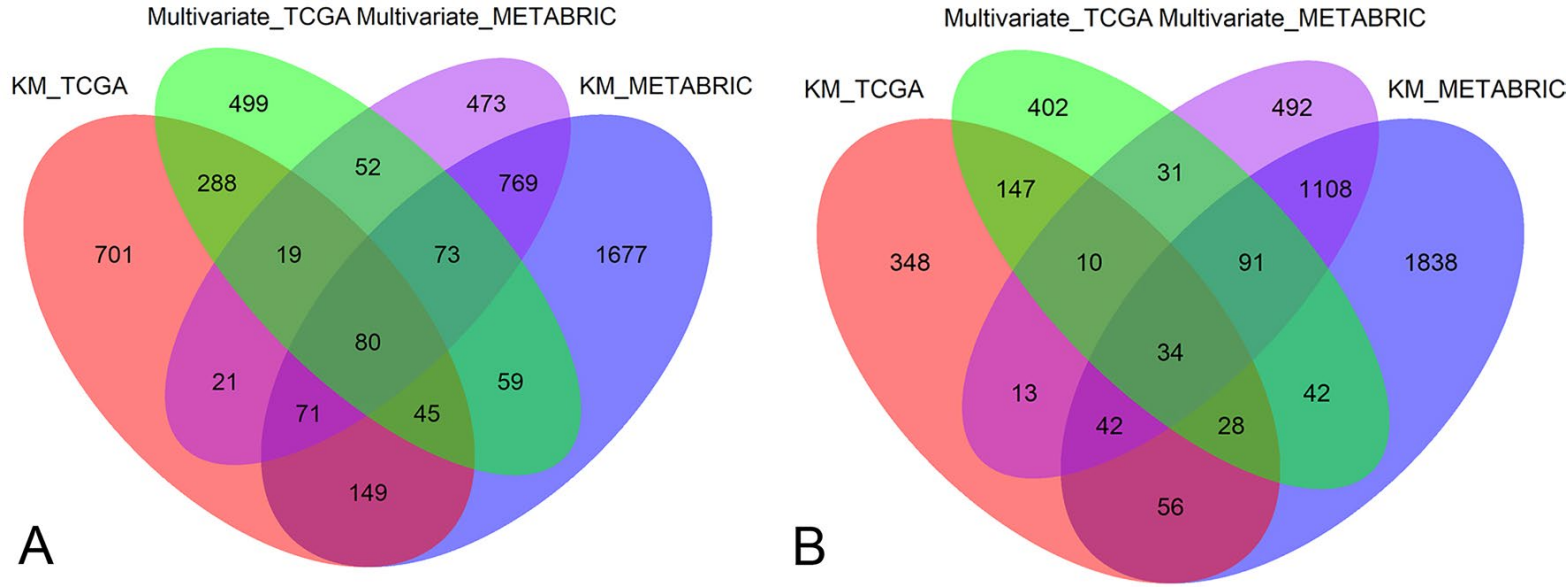
The prognosis-related genes common to the TCGA and METABRIC datasets. (**A**) The protective prognostic genes common to the TCGA and METABRIC datasets. (**B**) The risk prognostic genes common to the TCGA and METABRIC datasets. KM_TCGA and multivariate_TCGA represent prognosis-related genes determined by the Kaplan–Meier survival analysis and multivariate Cox regression analysis respectively in the TCGA cohort. Similarly, KM_METABRIC and multivariate_METABRIC denote survival-related genes in the METABRIC cohort.

We first examined the relation between gene expression and OS in the TCGA data set. The results showed that high expression levels of 1374 genes were related to significantly prolonged OS. While, high expression levels of 678 genes were related to significantly reduced survival in the TCGA cohort (P < 0.05 for all cases, log rank test, Fig. 1). Multivariate Cox regression analysis confirmed 432 protective prognostic genes and 219 risk prognostic genes following the adjustment of clinical characteristics. Furthermore, the association between 651 gene expression and OS was analyzed in the METABRIC dataset (n = 1904). The results validated 80 protective genes and 34 risk genes in the METABRIC cohort respectively (P < 0.05 for all cases, log rank test, Fig. 1). Then, we analyzed the functional involvement of the protective and risk genes with g.profiler and uncovered the 80 protective genes were significantly enriched in the KEGG pathway of focal adhesion. While, the risk genes were significantly over-represented in GO terms, such as nuclear division, organelle fission, DNA metabolic process and nucleic acid metabolic process (adjusted P value < 0.05 for all cases, supplementary Fig. 1).

### Construction of a 16-gene signature and its prognostic value in BRCA

**Figure 2:**
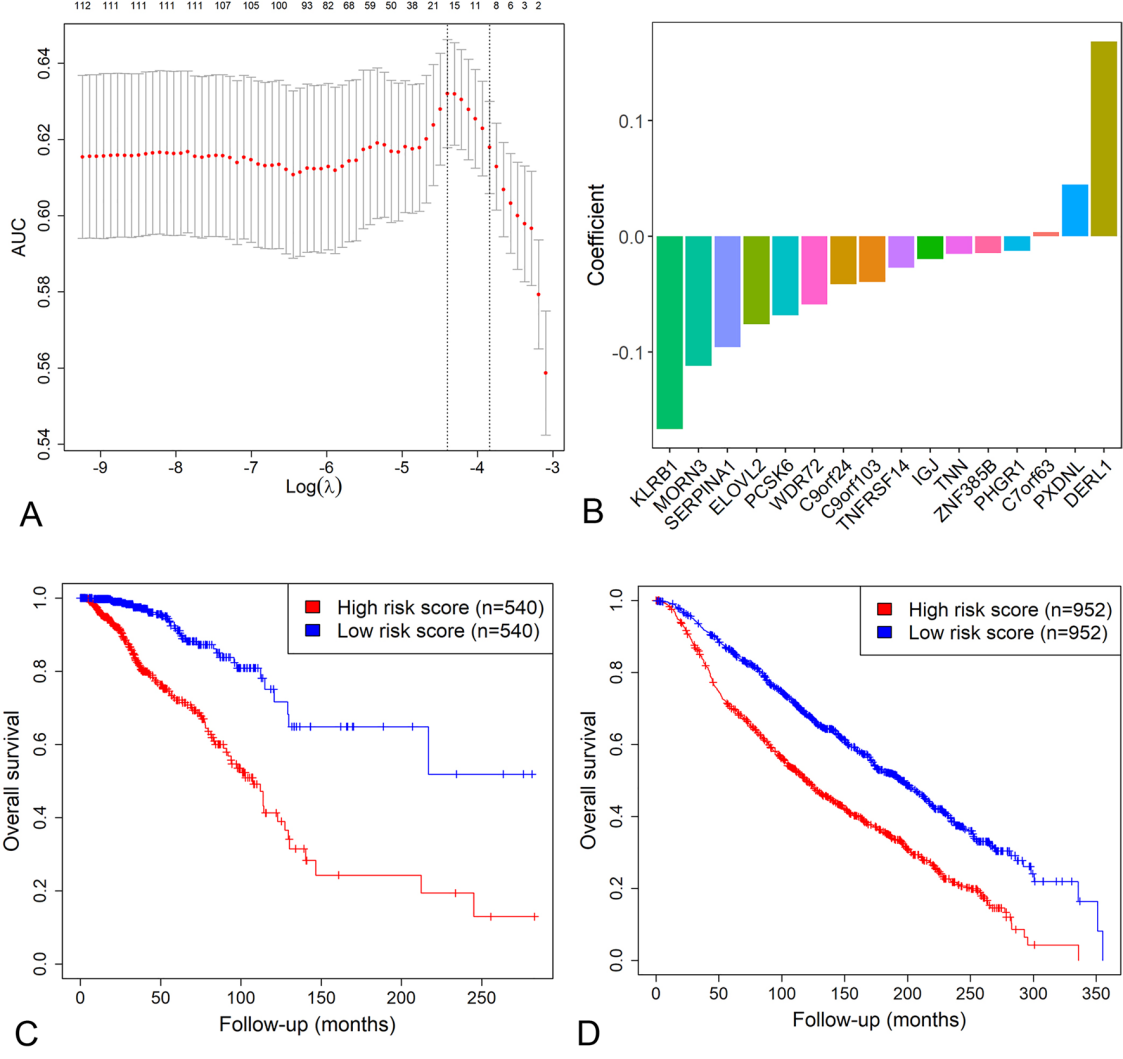
The 16-gene score is an indicator of infavorable survival in BRCA. (**A**) The relationship among AUC, log scaled lambda values and number of genes with non-zero coefficients in the LASSO model. The x axis represents the log value of the independent variable λ, whilst the y axis represents the partial likelihood deviance of the log value of each independent variable λ. (**B**) The coefficients of 16 genes in the LASSO model. (**C**) Kaplan–Meier survival curve of patients’ OS for BRCA patients with different 16-gene scores in the TCGA cohort, (**D**) Kaplan–Meier survival curve of patients’ OS for BRCA patients with different 16-gene scores in the METABRIC dataset.

We followed Lai et al.’s methods^15^ to choose the set of genes which performed best in prognosis prediction and develop the 16-gene risk score from 114 selected genes. The LASSO model comprising 16 genes showed the highest AUC value and was deemed the best model for survival prediction (Fig. 2A). Then we established the 16-gene score formula and computed the risk score for each BRCA patient, the coefficients of the 16 genes were presented in Fig. 2B. The Kaplan–Meier survival analysis and multivariate Cox regression analysis indicated that the high 16-gene score was indicative of worse OS in BRCA (P < 0.05 for all cases, OR: 3.47, 95% confidence interval: 2.08–5.78, Fig. 2C and supplementary Fig. 2A). We also analyzed the association between the 16-gene score and DFS and PFS in the TCGA cohort. Similarly, we demonstrated that the high 16-gene score was significantly associated with shorter DFS and PFS (P value < 0.05 for all cases, supplementary Fig. 3). For further verification, the 16-gene score was calculated in the METABRIC dataset. The results also confirmed the negative correlation between the 16-gene score and patient's OS (Fig. 2D, supplementary Fig. 2B). Furthermore, the 16-gene score (AUC = 0.72, 0.71, 0.73, respectively) outperformed cancer stage (AUC = 0.71, 0.69, 0.66, respectively, supplementary Fig. 4) in predicting 1-year survival, 3-year survival and 5-year survival in the TCGA cohort. The results were also validated in the METABRIC cohort (supplementary Fig. 4) and suggested the 16-gene score is superior to cancer stage in the prediction of prognosis of BRCA patients. We also obtained 2913 breast cancer patients from the GSE202203 dataset to validate the association between gene expression, risk score and overall survival. As expected, 12 of the ten survival-related genes showed positive correlation with prolonged survival and functioned as protective genes, *PXDNL* and *DERL1* were shown as risk gene (supplementary Table 1). Furthermore, we recalculated risk score using the validation dataset and confirmed the risk score is a negative factor for overall survival in breast cancer (supplementary Fig. 5).

### Correlations between the 16-gene score and clinical factors in BRCA

**Figure 3:**
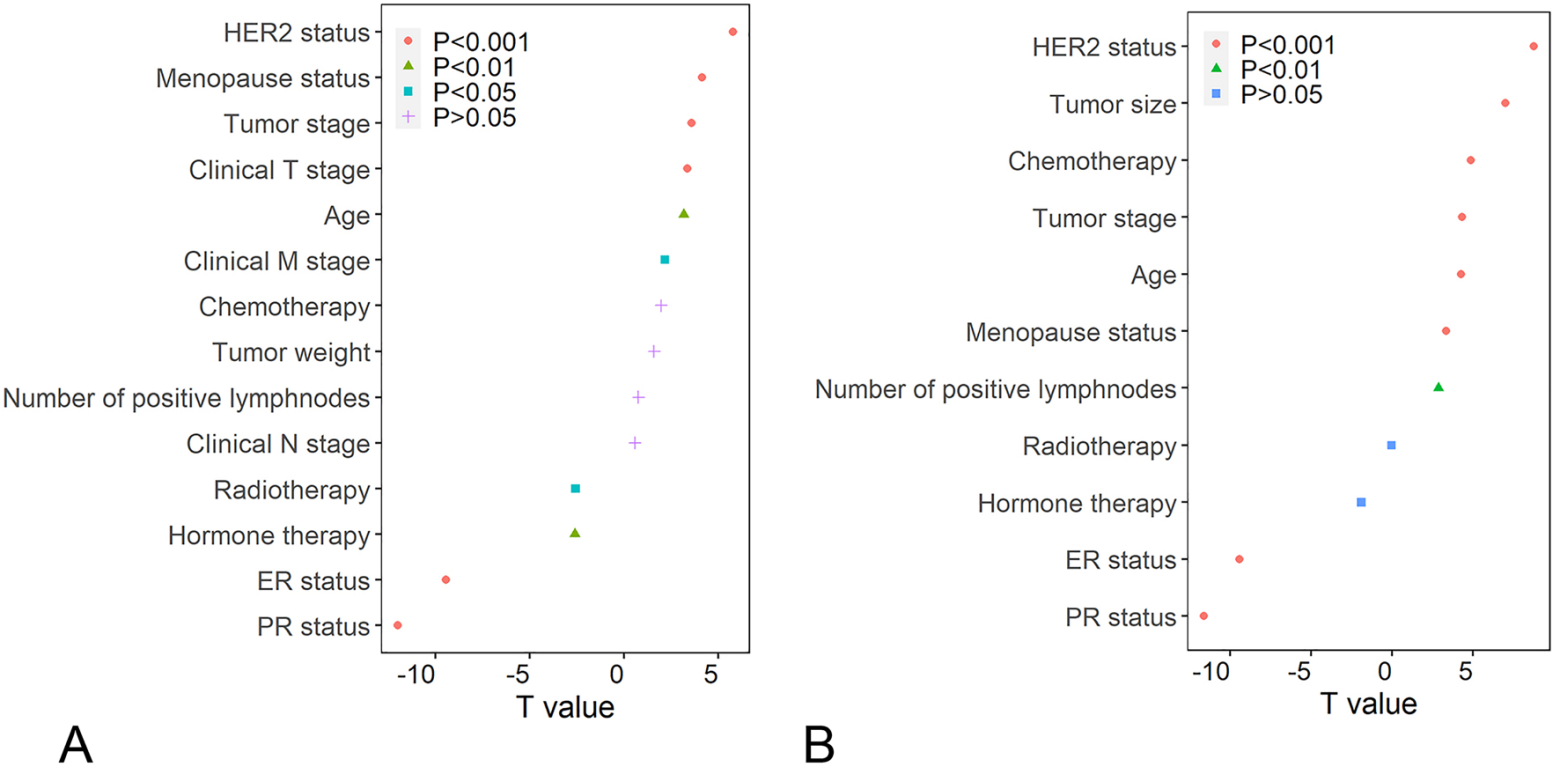
The correlations between the clinical characteristics and the 16-gene score. (**A**) The associations between clinical characteristics and the 16-gene score in the TCGA cohort. (**B**) The associations between clinical characteristics and the 16-gene score in the METABRIC cohort.

The linear regression model analysis showed the 16-gene score was significantly positively associated with age, HER2 status, menopause status, clinical stage, clinical T stage, clinical M stage and negatively correlated with PR status, ER status, hormone therapy and radiotherapy in the TCGA cohort (p < 0.05 for all cases, Fig. 3A). Moreover, the 16-gene score also exhibited positive correlation with age, HER2 status, menopause status, clinical stage and negative correlation with PR status, ER status, hormone therapy and radiotherapy in the METABRIC cohort (p < 0.05 for all cases, Fig. 3B). Next, we split BRCA patients into subgroups according to the clinical characteristics and conducted the Kaplan–Meier survival analysis to assess the prognostic value of the 16-gene score in clinical factor-specific subgroups. Overall, the results demonstrated that the high-risk was significantly correlated with worse OS in the same clinical subgroup of the TCGA cohort (P < 0.05 for all cases, log rank test, supplementary Table 2 and supplementary Fig. 6). Similar findings were also observed in the METABRIC cohort (supplementary Table 3 and supplementary Fig. 7), suggesting that the implication of 16-gene score with OS is independent of clinicopathological characteristics.

### Identification of signalling pathways associated with the 16-gene score

**Figure 4:**
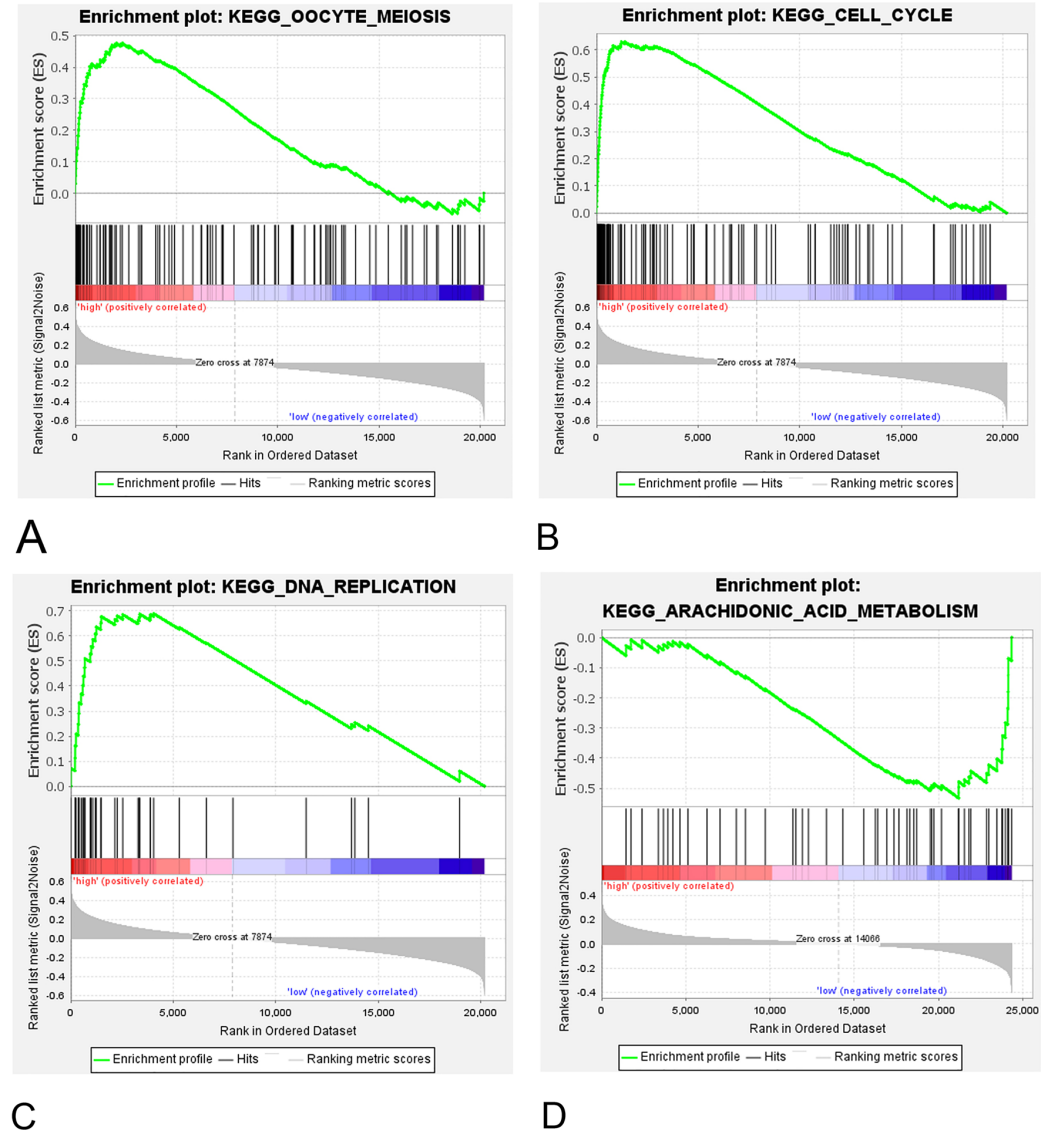
GSEA analysis revealed three significantly enriched pathways related to the high 16-gene score, including oocyte meiosis (**A**), cell cycle (**B**), DNA replication (**C**), and the significantly up-regulated arachidonic acid metabolism (**D**) associated with the low 16-gene score. For each gene set, vertical bars along the x-axis represent where the genes locate within the ranked list. Negative enrichment score indicates down-regulation, while, positive value denotes up-regulation of the gene set.

We performed the GSEA analysis to understand the biological functions related to the 16-gene score. The results exhibited thirteen signalling pathways were significantly over-represented in the high 16-gene score group of the TCGA cohort. Cell cycle, RNA degradation, oocyte meiosis, progesterone mediated oocyte maturation and DNA replication were the top five most enriched pathways (Fig. 4, q value < 0.25 for all cases, supplementary Table 4). While, up-regulation of arachidonic acid metabolism pathway genes were significantly associated with the low 16-gene score in the TCGA cohort (Fig. 4, q value < 0.25, supplementary Table 5). These results suggest that the aforementioned pathways probably are implicated in the association between 16-gene score and OS in BRCA.

### Nomogram combined 16-gene signature and clinical‐related variables predicts patients’ OS

**Figure 5:**
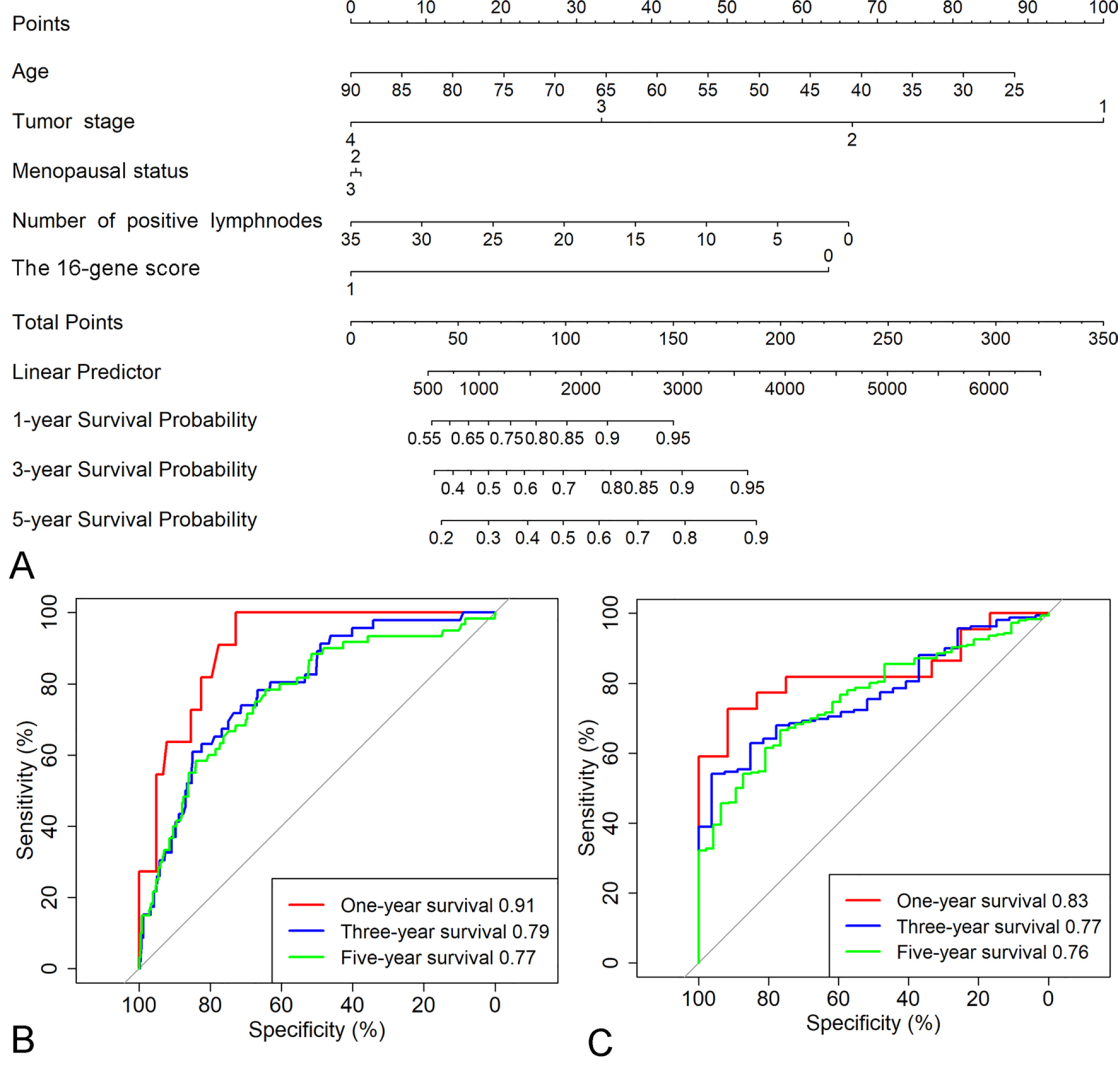
The 16-gene nomogram to predict the risk of disease in patients with BRCA. (**A**) mRNA nomogram to predict disease‐free survival. 1, 2, 3 for the menopausal status denote pre-menopause, peri-menopause and post-menopause respectively. 0 and 1 for the 16-gene score represent high and low 16-gene scores respectively which were divided by the median 16-gene score. (**B**) The ROC plot for the nomogram in predicting of 1-year, 3-year and 5-year survival in the TCGA cohort. (**C**) The ROC plot for the nomogram in predicting of 1-year, 3-year and 5-year survival in the METABRIC cohort.

In the TCGA and METABRIC cohorts, patient’s age, tumor stage, menopause status, number of positive lymph nodes and 16-gene signature were significantly associated with OS. Then based on the above analysis results, we established a 16-gene nomogram that incorporated the survival‐related clinical factors and 16-gene signature (Fig. 5A). The nomogram predicted well the 1-year, 3-year and 5‐year survival for BRCA patients in the TCGA cohort, ROC plot revealed the 16-gene nomogram showed improved prediction accuracies for 1-year, 3-year and 5‐year survival as compared to the 16-gene score alone (AUC: 0.91, 0.79 and 0.77 respectively, Fig. 5B). The improved prognosis prediction was also validated in the METABRIC cohort (AUC: 0.83, 0.77 and 0.76 respectively, Fig. 5C), demonstrating the clinical value and validity of the 16-gene nomogram for OS evaluation of BRCA patients.

### Assessment of diagnostic value

**Figure 6:**
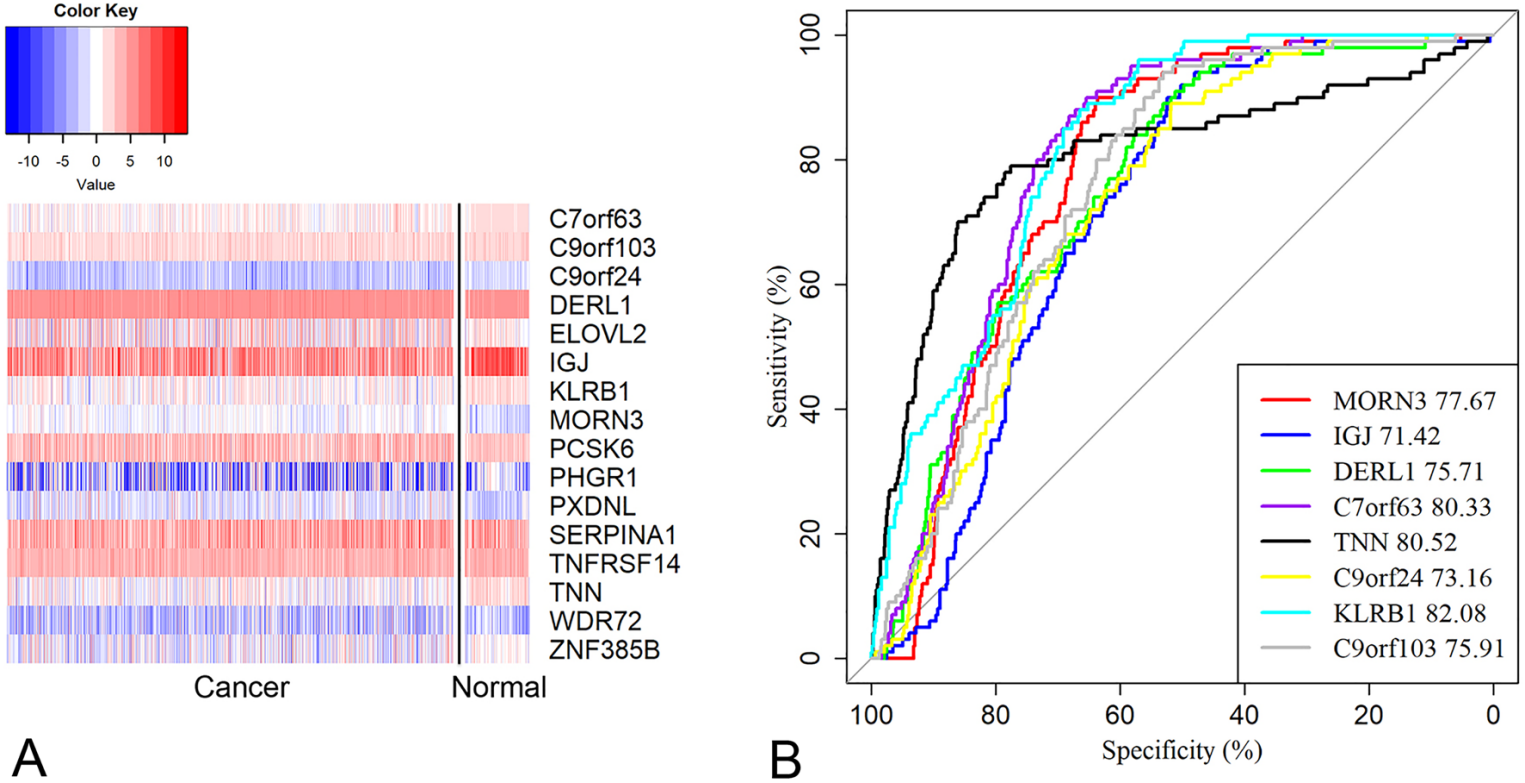
Gene expression analysis of prognosis-related genes. (**A**) The heatmap shows the expression differences of 16 prognosis-related genes between BRCA tissues and paired normal breast tissues. (**B**) The ROC curves for the top eight prognosis-related genes showing the highest diagnostic capability.

We utilized the online server cBioPortal to investigate the genomics variants of 16 genes from the TCGA datasets. The results showed that *DERL1, TNN, PXDNL, PCSK6* and *KLRB1*were the top five most frequently mutated genes, with mutation frequencies of 19%,10%, 9% 4%, 3% respectively in BRCA (supplementary Fig. 8). Similar mutation distribution was observed in the METABRIC cohort (supplementary Fig. 9). By comparing expression levels of 16 genes between 779 BRCA samples and 100 paired normal breast tissues, 7 genes expression, such as *C7orf63, C9orf103, IGJ, ZNF385B* and *TNN,* was significantly lower in tumor tissues as compared with those in normal tissues. In contrast, 9 genes, such as *PXDNL, PCSK6, MORN3* and *DERL1*, were significantly higher expressed in BRCA tissues (adjusted P < 0.05 for all cases, student t test, Fig. 6A). ROC curves analysis further showed *MORN3, IGJ, DERL1* particularly were able to differentiate BRCA tissues from normal breast tissues with high accuracy (Fig. 6B, adjusted P values < 0.05, AUC > 0.80 for all cases).

## Discussion

BRCA is a heterogeneous disease with several molecular subtypes, each of which has its distinct biological and clinical characteristics^19^. The identification of reliable prognostic biomarkers would enable to prioritize patients at high risk for death and relapse and guide treatment. The traditional methods for the risk stratification include tumor size, tumor stage, lymph node metastasis and molecular subtype, which could be applicable to certain subgroup of BRCA, however, there is still lack of a prognostic model that could be applicable to almost all BRCA subtypes. Recent studies have shown gene expression profiles could serve as prognostic biomarkers in BRCA^20,21^. However, the accuracies of the previous gene profiles are still relatively low. In the current study, we have successfully established the 16-gene score which is correlated with poor OS, DFS and PRS in BRCA. We also demonstrated that the prognostic value of the 16-gene score was independent of clinical factors and applied to all subtypes of BRCA patients, which is advantageous to the MammaPrint model and Oncotype DX that show applicability to limited disease subtypes. Furthermore, we established a 16-gene nomogram that incorporated the survival‐related clinical factors and 16-gene signature. As compared to established gene profiles, the 16-gene nomogram (AUC = 0.91, 0.79 and 0.77, respectively) performed better than the Teschendorff's^22^ (AUC = 0.44, 0.47, 0.50, respectively) and Bianchini's^23^ immune-related gene signatures (AUC = 0.53, 0.56, 0.51, respectively)^20^ and cancer stage (AUC = 0.71, 0.69, 0.66, respectively) in predicting the 1-year, 3-year and 5-year survival of BRCA patients. Therefore, the 16-gene nomogram might be a reliable and useful prognostic tool for OS evaluation and will promote tailored therapy for all subtypes of BRCA patients.

The mechanisms by which the higher 16-gene score is associated with poor prognostic implication remain to be poorly understood. The GESA analysis uncovered cell cycle, RNA degradation, oocyte meiosis, progesterone mediated oocyte maturation and DNA replication were significantly over-represented in the high 16-gene score group. Cell cycle checkpoints are critical for ordered cell cycle progression, which ensures genomic stability and inhibits the process of carcinogenesis^24^. The deregulation of the cyclin-dependent kinase inhibitors p21 and p27, cyclins D1 and E frequently exerts negative impacts on BRCA outcome and response to therapy^25^. We believe the dysregulation of cell cycle signalling pathway largely contribute to the prognostic value of 16-gene score in BRCA.

Of the 16 prognosis-related genes, many have been shown to play key roles in the development and progression of various cancers. High expression of *SERPINA1* gene encoding acute phase protein, alpha1-antitrypsin (AAT), is associated with various tumors. Experiments in vitro revealed that external AAT and/or overexpressed *SERPINA1* gene significantly enhanced cancer cell migration, colony formation and resistance to apoptosis^26^. The *SERPINA1* gene functions as key prognostic gene for patients with colon cancer^27^ and non-small-cell lung cancer patients^26^. *SERPINA1* has also a significant predictive value for the OS of ER+/HER2+ patients. ER is constitutively activated, leading to an E2-independent ER binding to the SERPINA1 gene and upregulation of SERPINA1 expression in breast cancer^28^. *KLRB1* is encoded by killer cell lectin-like receptor B1 gene and a new candidate inhibitor of tumour-infiltrating T cells. *KLRB1* was differentially expressed and associated with better overall survival in a variety of tumour types. In addition, *KLRB1* expression was significantly associated with immunoregulatory interactions between lymphoid and non-lymphoid cells, T cell infiltration, immune checkpoints, immune activating genes, immunosuppressive genes, chemokines, and chemokine receptors^29^. *KLRB1* (coding for CD161) gene expression shows a positive association with favorable outcome in non-small-cell lung cancer, independently of the size of T and B cell infiltrates, making CD161-expressing CD4^+^ T cells ideal candidates for anti-tumor recall responses^30^. Elongation of very-long chain fatty acid protein 2 (ELOVL2) was hypermethylated and downregulated in tamoxifen-resistant breast cancer patients as compared with the tamoxifen-sensitive patients. *ELOVL2* was shown to increase tamoxifen sensitivity up to 70% in the MCF-7/tamoxifen-resistant cells and in a xenograft mouse model^31^. Of note, elevated *ELOVL2* expression levels were observed in renal cell carcinoma and significantly associated with a poor prognosis of patients with renal cell carcinoma. Furthermore, *ELOVL2* promotes cancer progression by repressing cell apoptosis in renal cell carcinoma^32^.

Though we have established a risk score that is predictive of overall survival of BRCA patients independently of clinical characteristics, this study shows a number of limitations. First of all, the prediction accuracy of the nomogram incorporating clinical factors and risk scores is relatively low for 3-year and 5‐year survival, therefore, future studies may need to add more useful features to increase the performance of our model. Secondly, the functions of the identified prognosis-associated genes have not been fully illustrated, more experimental investigation should be performed to further characterize their biological functions in breast cancer.

## Conclusion

Taken together, this study identified a novel 16 gene signature that could serve as an independent factor for predicting BRCA prognosis independently of clinical characteristics. The gene set related to the high-risk group participated in the cell cycle signal pathway.

## Supplementary Information

Supplementary Figures.

Supplementary Tables.

## Data Availability

The datasets generated and/or analysed during the current study are available in the figshare repository (figshare ID: 15048003, https://figshare.com/s/df0ee21997f1aa0da4bd).

## Code availability

The codes in the current study are available upon reasonable request.
