# Supplementary material for: Identification and validation of a novel 16-gene prognostic signature for patients with breast cancer
Source: Sci Rep. 2022 Jul 19;12:12349. doi: 10.1038/s41598-022-16575-8 (PMC9296560; doi:10.1038/s41598-022-16575-8)
Supplement: Supplementary file 1 — Supplementary Figures. [file 41598_2022_16575_MOESM1_ESM.docx]

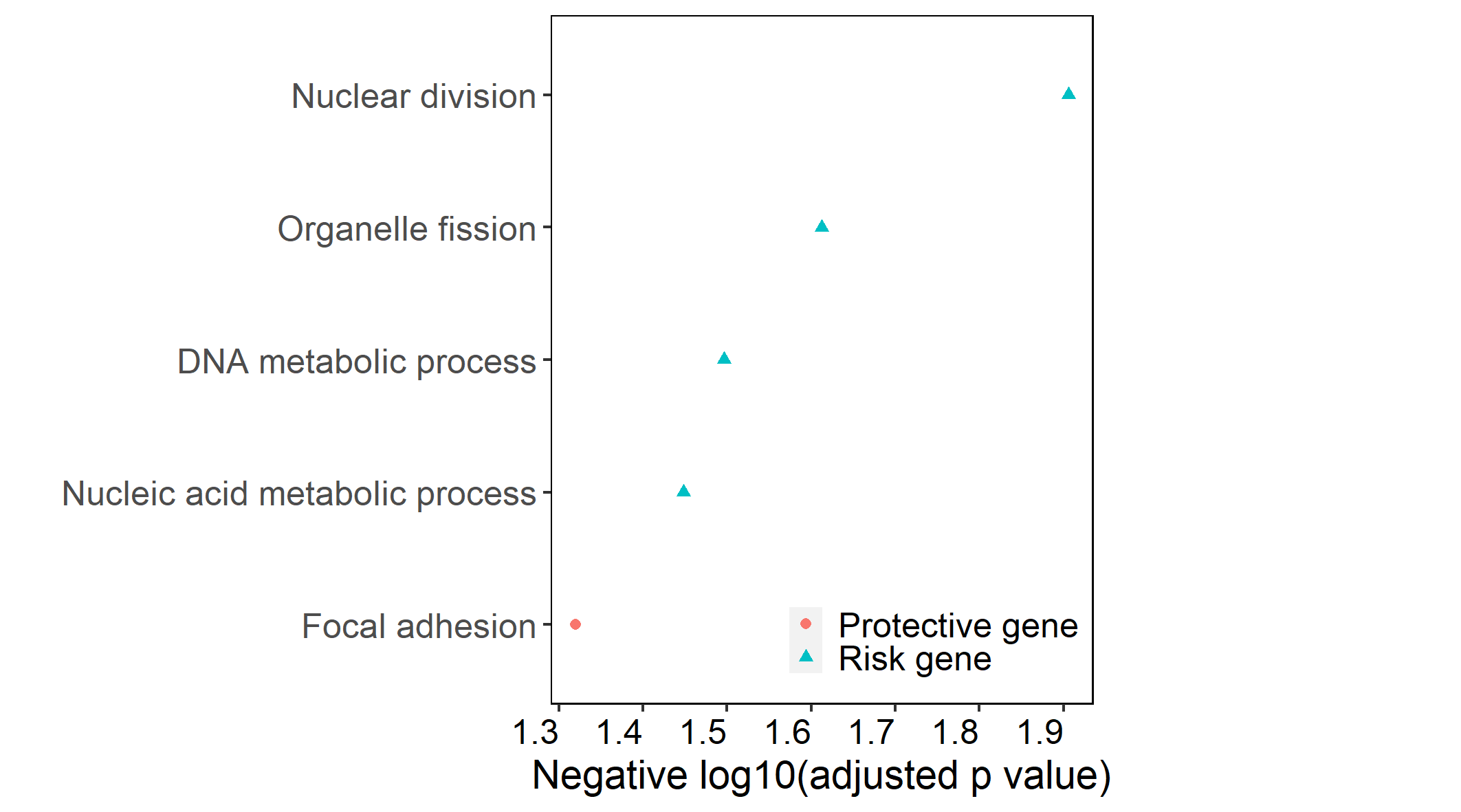


Supplementary Figure1. The GO terms and KEGG pathways significantly enriched for protective prognostic genes and risk genes.


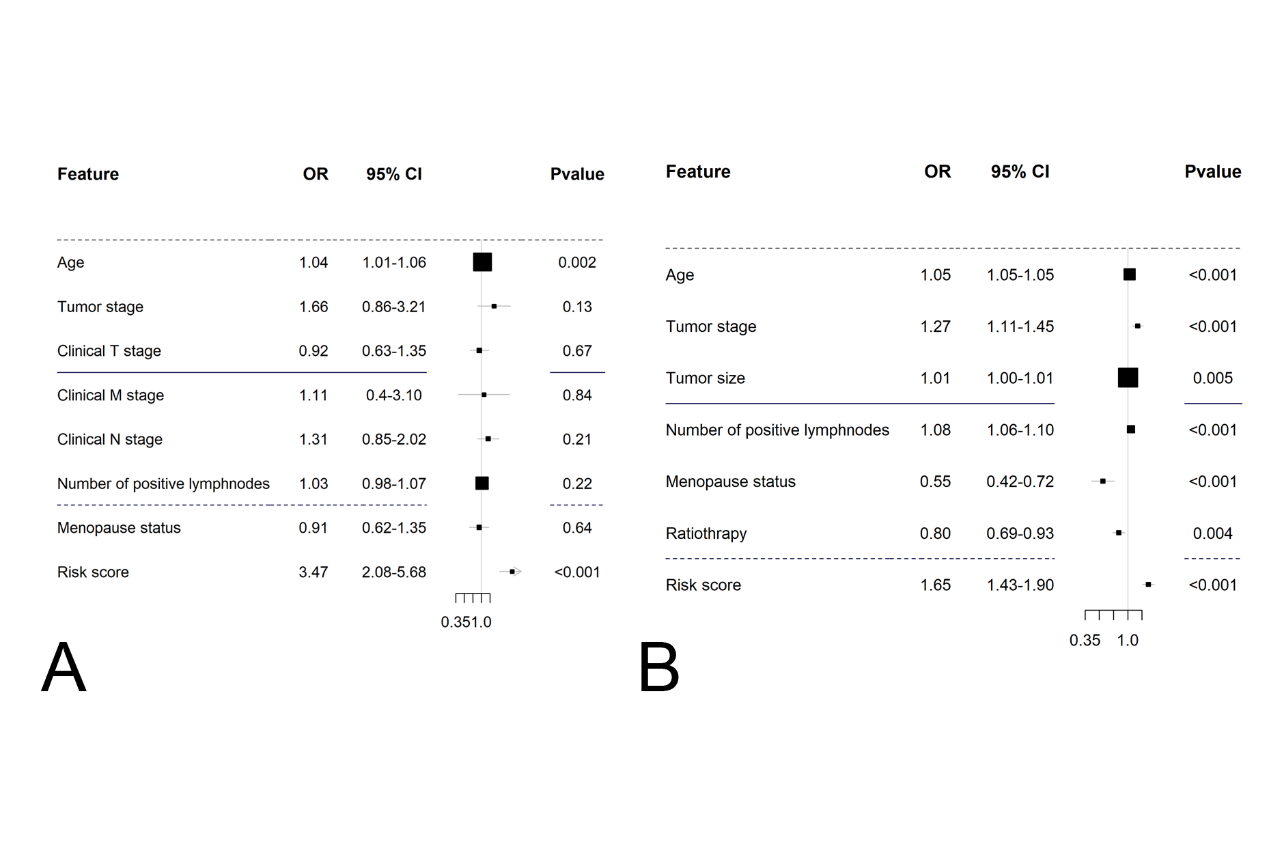


Supplementary Figure2. The forest plot constructed based on the 16-gene score values and the clinical features in the TCGA (A) and METABRIC (B) cohorts. OR, Odd ratio, CI, confidence interval.


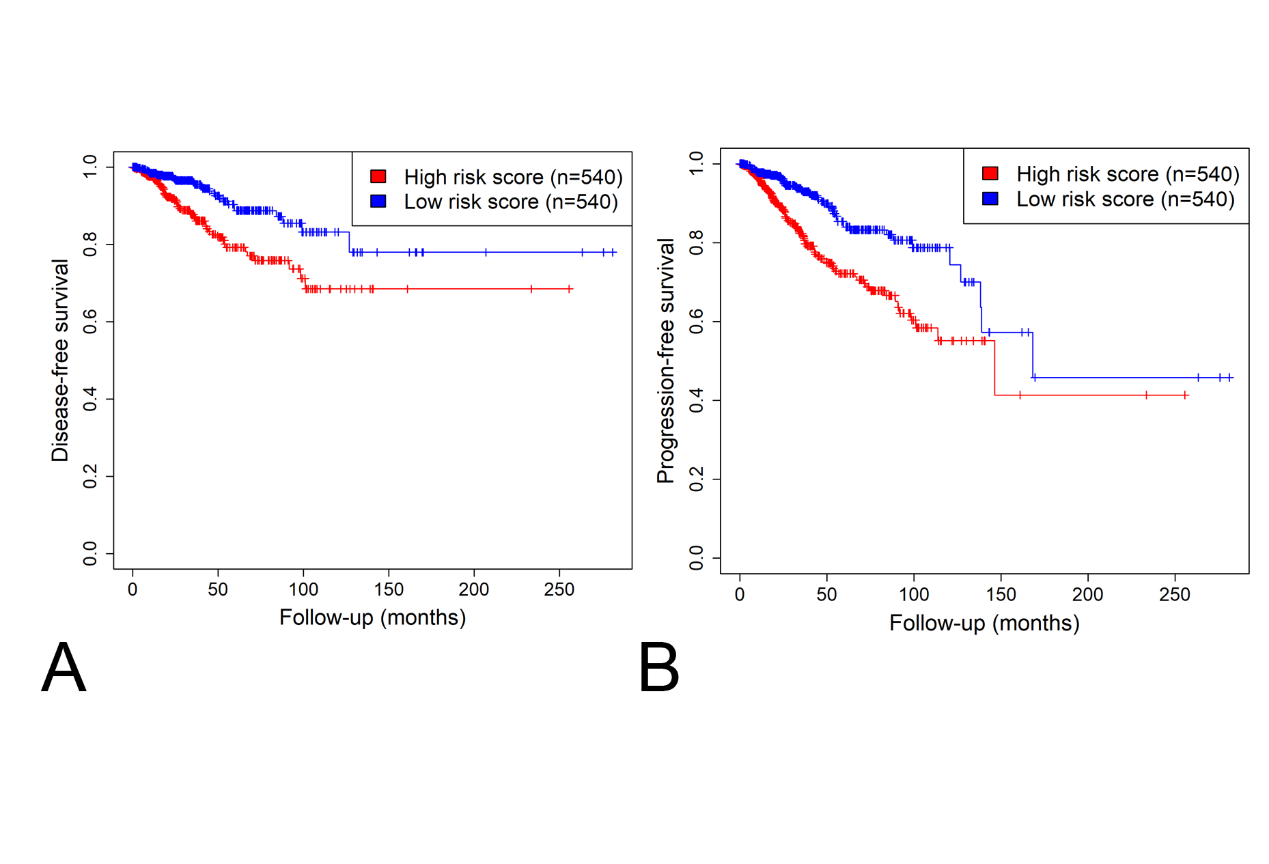


Supplementary Figure3. Kaplan-Meier curves of DFS (A) and PFS (B) for BRCA patients with different 16-gene scores in the TCGA cohort.


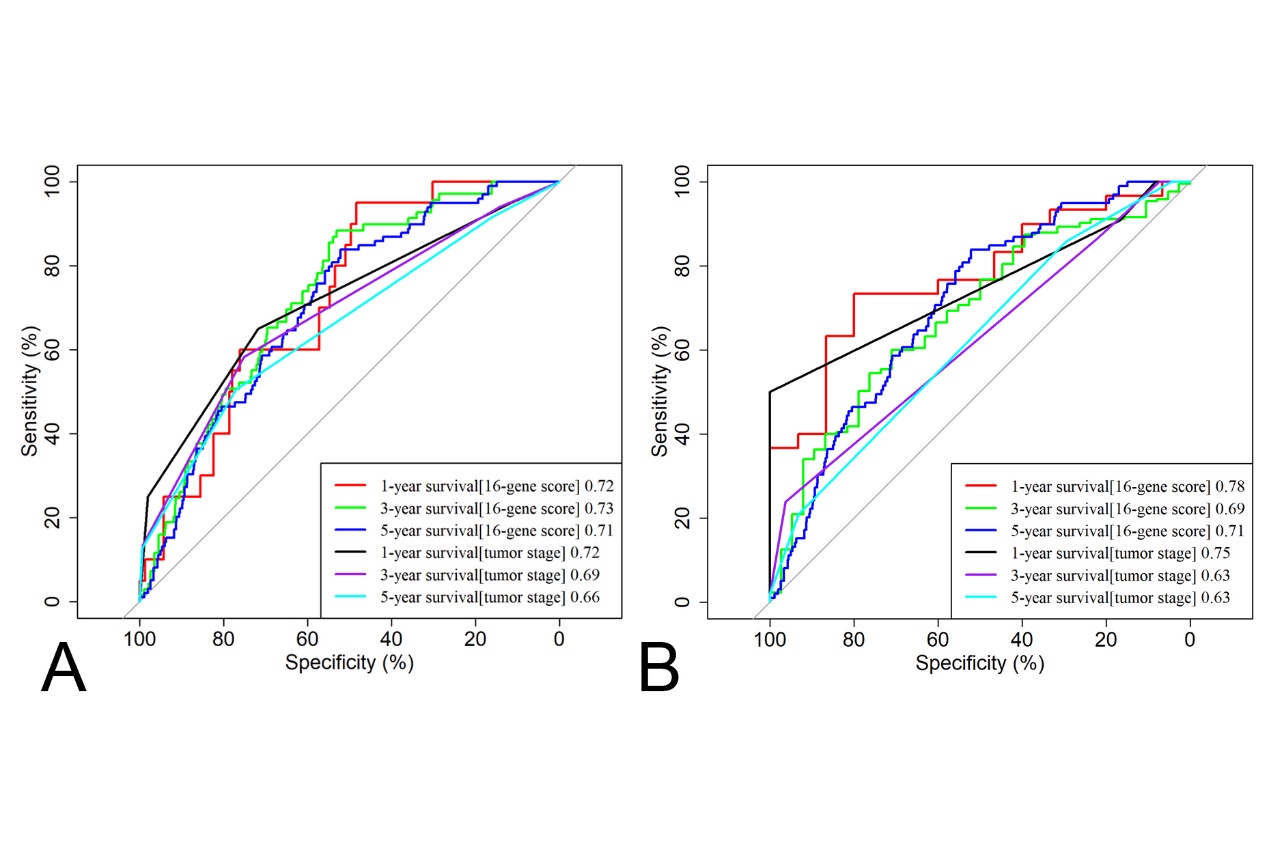


Supplementary Figure4. The ROC curves for the 16-gene score and cancer stage in the TCGA and METABRIC datasets.


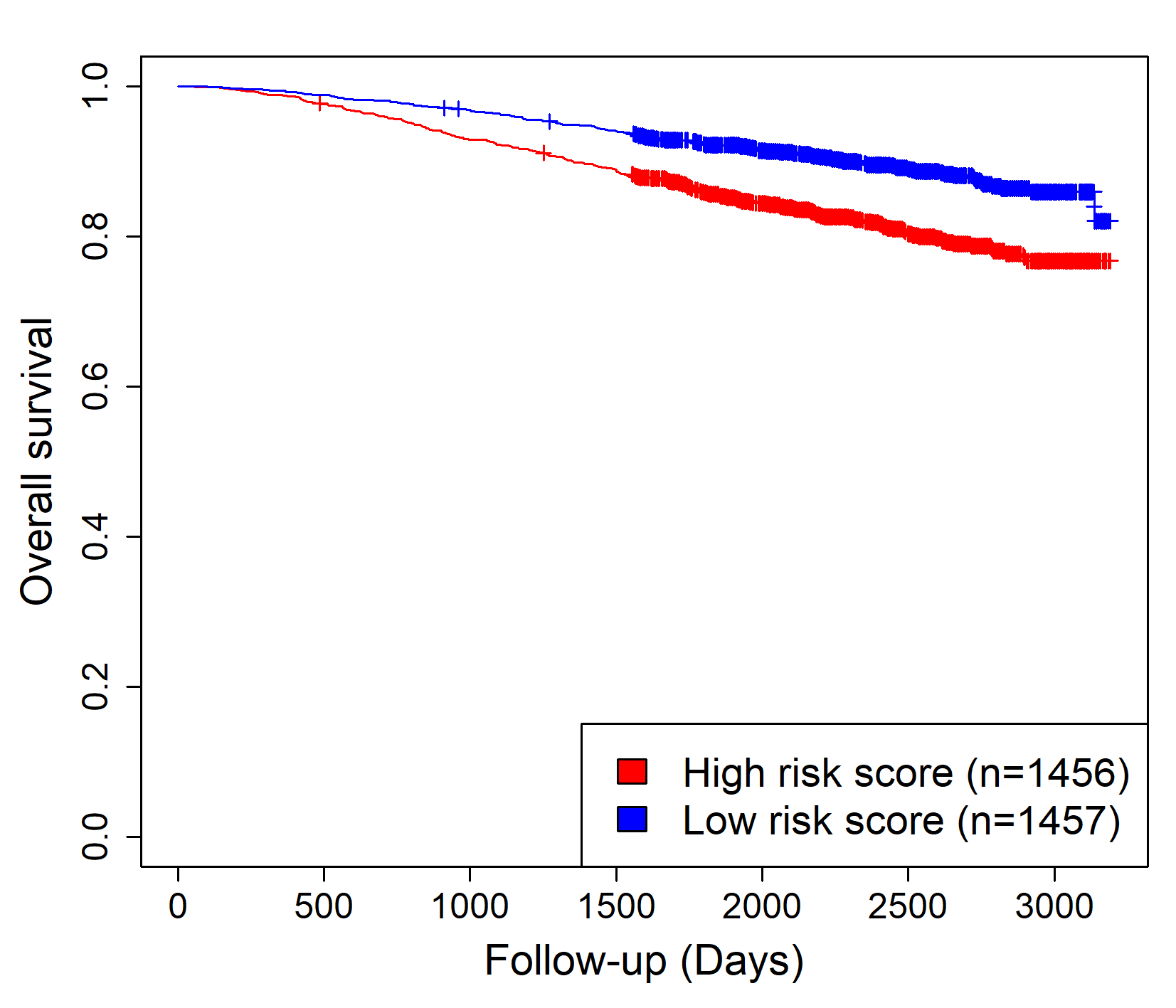
Supplementary Figure5. Kaplan-Meier survival analysis shows the negative correlation between risk score and overall survival in BRCA patients of the GSE202203 dataset.


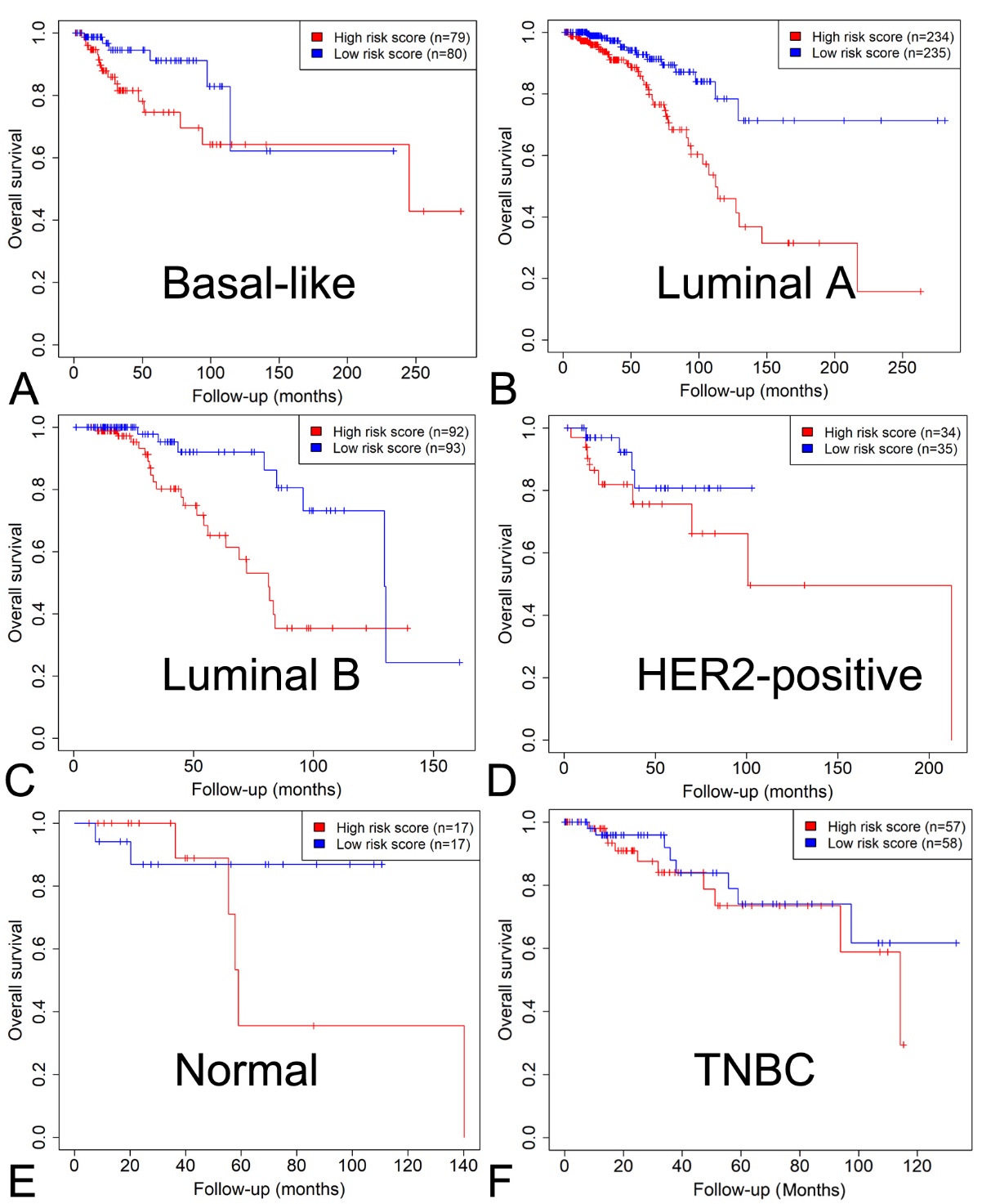


Supplementary Figure6. Kaplan-Meier survival analysis shows the negative correlation between risk score and overall survival in different subtypes of BRCA patients, including Basal-like, Luminal A, Luminal B, HER2-positive, Normal and Triple negative breast cancer (TNBC) (A-F) subtypes in the TCGA cohort.


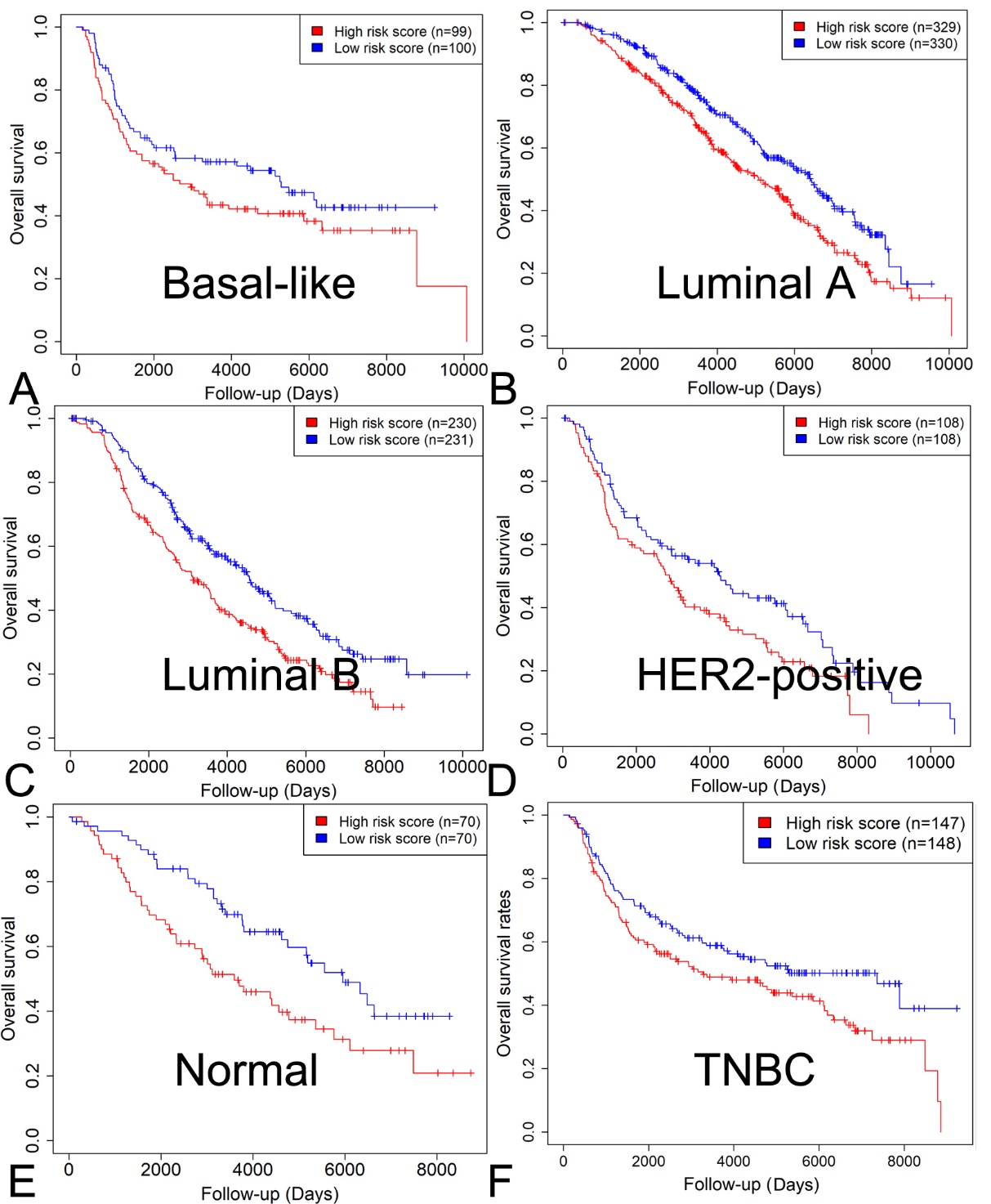


Supplementary Figure7. Kaplan-Meier survival analysis shows the negative correlation between risk score and overall survival in different subtypes of BRCA patients, including Basal-like, Luminal A, Luminal B, HER2-positive, Normal and Triple negative breast cancer (TNBC) (A-F) subtypes in the METABRIC cohort.


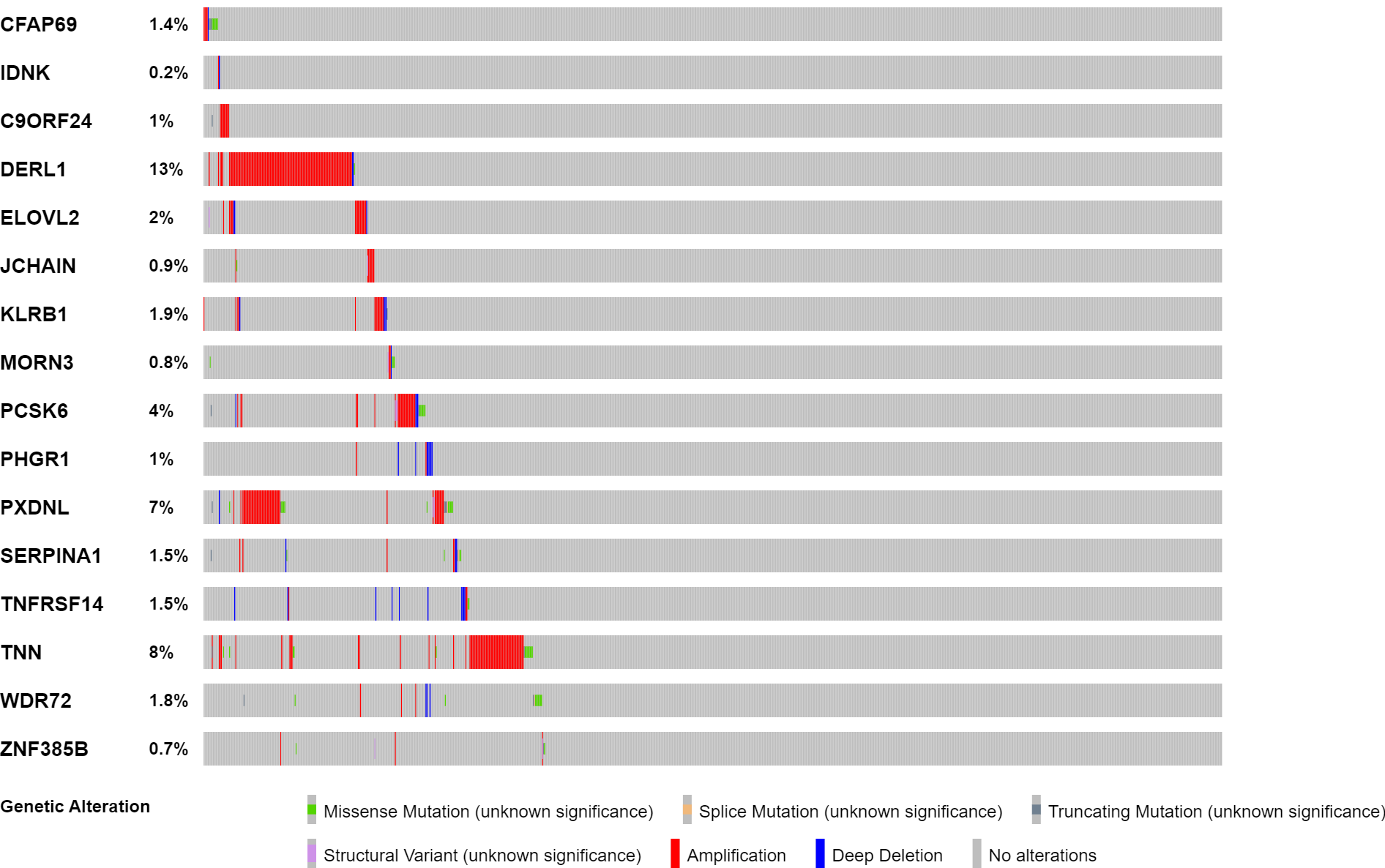
Supplementary Figure8. The mutational profiles for the 16 prognosis-related genes in the TCGA dataset.


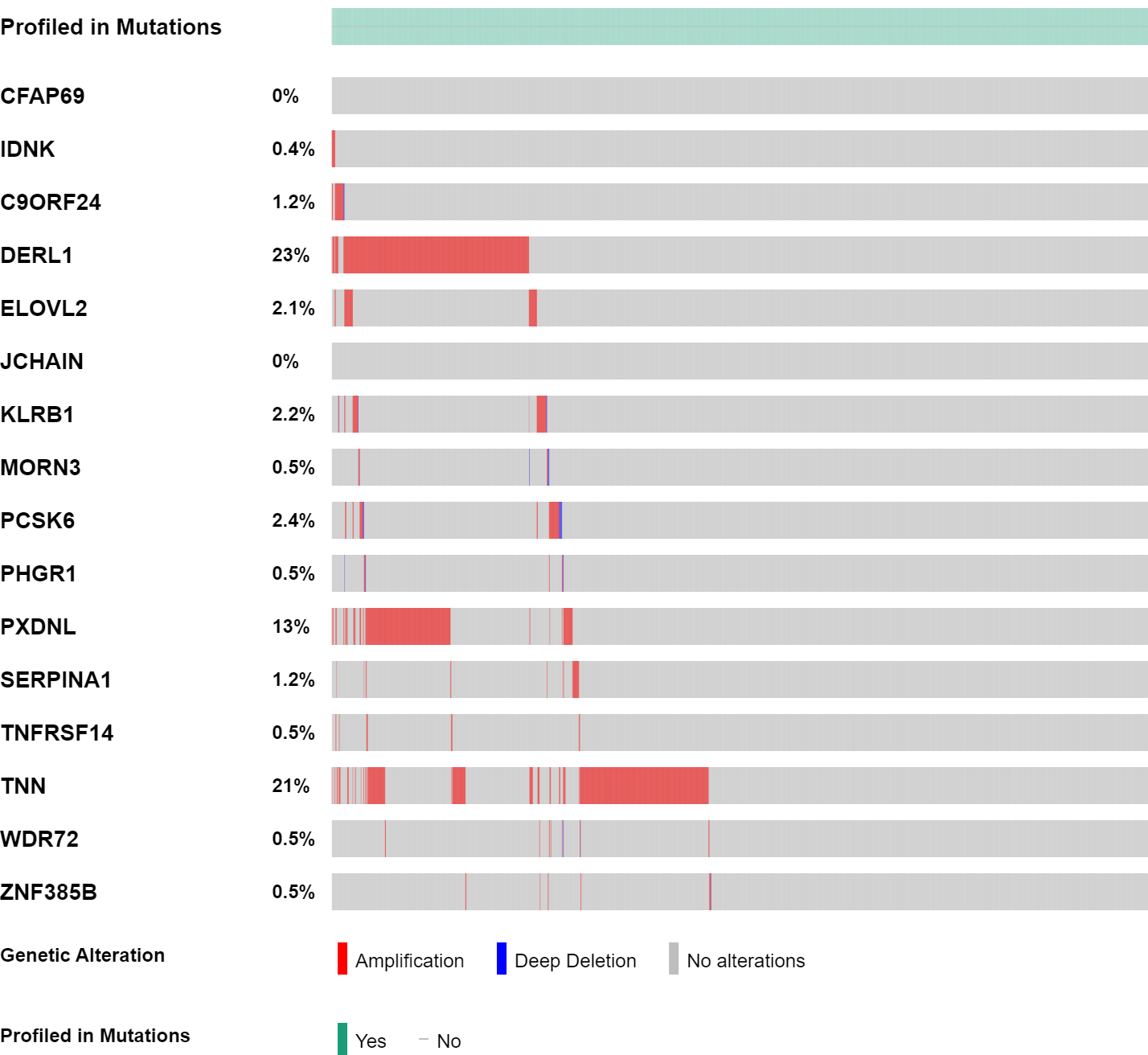


Supplementary Figure9. The mutational profiles for the 16 prognosis-related genes in the METABRIC dataset.
