# Supplementary material for: Identification and validation of a novel 16-gene prognostic signature for patients with breast cancer
Source: Sci Rep. 2022 Jul 19;12:12349. doi: 10.1038/s41598-022-16575-8 (PMC9296560; doi:10.1038/s41598-022-16575-8)
Supplement: Supplementary file 2 — Supplementary Tables. [file 41598_2022_16575_MOESM2_ESM.docx]

Supplementary table1. The Kaplan-Meier survival analysis results between the 16-gene score and overall survival in the BRCA patients of the GSE202203 dataset.

| Gene | Chistq value | P value | Correlation |
| --- | --- | --- | --- |
| C9orf24 | 0.58 | 0.45 | Positive |
| DERL1 | 4.67 | 0.03 | Negative |
| ELOVL2 | 42.32 | <0.001 | Positive |
| KLRB1 | 27.11 | <0.001 | Positive |
| MORN3 | 6.43 | 0.01 | Positive |
| PCSK6 | 29.09 | <0.001 | Positive |
| PHGR1 | 26.18 | <0.001 | Positive |
| PXDNL | 8.79 | <0.001 | Negative |
| SERPINA1 | 0.96 | 0.33 | Positive |
| TNFRSF14 | 16.51 | <0.001 | Positive |
| TNN | 53.51 | <0.001 | Positive |
| WDR72 | 12.55 | <0.001 | Positive |
| ZNF385B | 37.62 | <0.001 | Positive |
| C9orf103 | 19.20 | <0.001 | Positive |
| IGJ | 39.91 | <0.001 | Positive |
| C7orf63 | 27.26 | <0.001 | Positive |

Supplementary table2. The Kaplan-Meier survival analysis results between the 16-gene score and overall survival in the BRCA patients stratified by clinical features of the TCGA cohort.

| Feature | Classification | Chistq value | P value |
| --- | --- | --- | --- |
| Age | <58 | 22.12 | <0.001 |
|  | >=58 | 22.38 | <0.001 |
| Tumor stage | I | 1.50 | 0.221 |
|  | II | 24.72 | <0.001 |
|  | III | 26.90 | <0.001 |
|  | IV | 0.94 | 0.333 |
| Clinical T stage | I | 1.91 | 0.167 |
|  | II | 26.66 | <0.001 |
|  | III | 9.27 | 0.002 |
|  | IV | 5.98 | 0.014 |
| Clinical M stage | 0 | 44.60 | <0.001 |
|  | 1 | 0.77 | 0.379 |
| Clinical N stage | 0 | 13.28 | <0.001 |
|  | 1 | 25.75 | <0.001 |
|  | 2 | 13.85 | <0.001 |
|  | 3 | 7.90 | 0.005 |
| Number of positive lymph nodes | >=1 | 43.18 | <0.001 |
|  | <1 | 10.01 | 0.002 |
| Menopause status | Pre-menopause | 13.78 | <0.001 |
|  | Post-menopause | 27.04 | <0.001 |
|  | Peri-menopause | 2.00 | 0.157 |
| ER status | Positive | 31.41 | <0.001 |
|  | Negative | 4.82 | 0.028 |
| HER2 status | Negative | 19.56 | <0.001 |
|  | Positive | 8.76 | 0.003 |
| PR status | Positive | 29.22 | <0.001 |
|  | Negative | 10.99 | 0.001 |
| Tumor weight | <218 | 23.24 | <0.001 |
|  | >=218 | 26.46 | <0.001 |
| Chemotherapy | Yes | 9.12 | 0.003 |
|  | No | 6.97 | 0.008 |
| Hormone therapy | No | 9.00 | 0.003 |
|  | Yes | 8.32 | 0.004 |
| Radiation therapy | No | 36.51 | <0.001 |
|  | Yes | 14.22 | <0.001 |
| Molecular subtype | Basal | 4.20 | 0.04 |
|  | LumA | 20.88 | <0.001 |
|  | LumB | 8.21 | 0.004 |
|  | Normal | 0.75 | 0.39 |
|  | Her2 | 1.58 | 0.22 |
|  | TNBC | 0.44 | 0.51 |

Supplementary table3. The Kaplan-Meier survival analysis results between the 16-gene score and overall survival in the BRCA patients stratified by clinical features of the METABRIC cohort.

| Feature | Classification | Chistq value | P value |
| --- | --- | --- | --- |
| Age | >=61.77 | 16.34 | <0.001 |
|  | <61.77 | 47.42 | <0.001 |
| Tumor stage | 0 | 0.00 | 1.000 |
|  | I | 14.72 | <0.001 |
|  | II | 36.79 | <0.001 |
|  | III | 0.12 | 0.726 |
|  | IV | 6.49 | 0.011 |
| Number of positive lymph nodes | >0 | 36.15 | <0.001 |
| Number of positive lymph nodes | =0 | 40.41 | <0.001 |
| Menopause status | Post-menopause | 37.99 | <0.001 |
|  | Pre-menopause | 25.92 | <0.001 |
| ER status | Positive | 67.99 | <0.001 |
|  | Negative | 13.96 | <0.001 |
| HER2 status | Negative | 54.98 | <0.001 |
|  | Positive | 5.58 | 0.018 |
| PR status | Negative | 16.99 | <0.001 |
|  | Positive | 43.00 | <0.001 |
| Tumor size | <23 | 13.20 | <0.001 |
|  | >=23 | 57.48 | <0.001 |
| Chemotherapy | No | 74.21 | <0.001 |
|  | Yes | 8.58 | 0.003 |
| Hormone therapy | Yes | 43.66 | <0.001 |
|  | No | 35.63 | <0.001 |
| Radiation therapy | Yes | 48.63 | <0.001 |
|  | No | 36.48 | <0.001 |
| Molecular subtype | LumA | 33.23 | <0.001 |
|  | LumB | 13.81 | <0.001 |
|  | Her2 | 6.50 | 0.01 |
|  | Normal | 6.69 | 0.01 |
|  | Basal | 2.37 | 0.12 |
|  | TNBC | 4.82 | 0.03 |

Supplementary table4. The significantly up-regulated signalling pathways in the high or low risk score group of the TCGA cohort

| KEGG pathway name | Pathway size | Enrichement score | P value | Q value |
| --- | --- | --- | --- | --- |
| *Gene sets enriched in phenotype high risk score* |  |  |  |  |
| KEGG_CELL_CYCLE | 118 | 0.63 | 0.00 | 0.04 |
| KEGG_OOCYTE_MEIOSIS | 110 | 0.48 | 0.00 | 0.18 |
| KEGG_RNA_DEGRADATION | 53 | 0.53 | 0.00 | 0.15 |
| KEGG_PROGESTERONE_MEDIATED_OOCYTE_MATURATION | 85 | 0.43 | 0.02 | 0.23 |
| KEGG_PROTEIN_EXPORT | 22 | 0.63 | 0.05 | 0.20 |
| KEGG_HOMOLOGOUS_RECOMBINATION | 24 | 0.62 | 0.05 | 0.22 |
| KEGG_RNA_POLYMERASE | 29 | 0.56 | 0.04 | 0.24 |
| KEGG_CYSTEINE_AND_METHIONINE_METABOLISM | 34 | 0.48 | 0.02 | 0.21 |
| KEGG_SPLICEOSOME | 110 | 0.49 | 0.06 | 0.23 |
| KEGG_MISMATCH_REPAIR | 23 | 0.60 | 0.06 | 0.21 |
| KEGG_PROTEASOME | 43 | 0.63 | 0.10 | 0.23 |
| KEGG_PYRIMIDINE_METABOLISM | 95 | 0.42 | 0.04 | 0.22 |
| KEGG_TERPENOID_BACKBONE_BIOSYNTHESIS | 15 | 0.60 | 0.08 | 0.24 |
|  |  |  |  |  |
| *Gene sets enriched in phenotype low risk score* |  |  |  |  |
| KEGG_ARACHIDONIC_ACID_METABOLISM | 55 | -0.55 | 0.00 | 0.20 |

Supplementary table5. The significantly up-regulated signalling pathways in the high or low risk score group of the METABRIC cohort

| KEGG pathway name | Pathway size | Enrichement score | P value | Q value |
| --- | --- | --- | --- | --- |
| *Gene sets enriched in phenotype high risk score* |  |  |  |  |
| KEGG_CELL_CYCLE | 122 | 0.63 | 0.00 | 0.00 |
| KEGG_BLADDER_CANCER | 38 | 0.60 | 0.00 | 0.02 |
| KEGG_HOMOLOGOUS_RECOMBINATION | 22 | 0.70 | 0.01 | 0.05 |
| KEGG_DNA_REPLICATION | 36 | 0.72 | 0.00 | 0.04 |
| KEGG_OOCYTE_MEIOSIS | 108 | 0.46 | 0.01 | 0.04 |
| KEGG_CYSTEINE_AND_METHIONINE_METABOLISM | 31 | 0.53 | 0.01 | 0.11 |
| KEGG_FRUCTOSE_AND_MANNOSE_METABOLISM | 33 | 0.51 | 0.02 | 0.13 |
| KEGG_RENAL_CELL_CARCINOMA | 62 | 0.44 | 0.01 | 0.15 |
| KEGG_VIBRIO_CHOLERAE_INFECTION | 52 | 0.46 | 0.01 | 0.13 |
| KEGG_PROGESTERONE_MEDIATED_OOCYTE_MATURATION | 82 | 0.42 | 0.01 | 0.12 |
| KEGG_BASE_EXCISION_REPAIR | 30 | 0.56 | 0.03 | 0.12 |
| KEGG_RNA_DEGRADATION | 52 | 0.48 | 0.02 | 0.12 |
| KEGG_UBIQUITIN_MEDIATED_PROTEOLYSIS | 124 | 0.39 | 0.01 | 0.12 |
| KEGG_SPLICEOSOME | 113 | 0.46 | 0.04 | 0.15 |
| KEGG_ERBB_SIGNALING_PATHWAY | 83 | 0.39 | 0.02 | 0.16 |
| KEGG_PYRIMIDINE_METABOLISM | 86 | 0.43 | 0.03 | 0.16 |
| KEGG_MISMATCH_REPAIR | 23 | 0.58 | 0.07 | 0.19 |
| KEGG_INSULIN_SIGNALING_PATHWAY | 132 | 0.34 | 0.01 | 0.19 |
| KEGG_ONE_CARBON_POOL_BY_FOLATE | 17 | 0.52 | 0.07 | 0.20 |
| KEGG_AMINOACYL_TRNA_BIOSYNTHESIS | 19 | 0.55 | 0.06 | 0.19 |
| KEGG_PURINE_METABOLISM | 139 | 0.35 | 0.03 | 0.20 |
| KEGG_GLYCEROPHOSPHOLIPID_METABOLISM | 66 | 0.37 | 0.03 | 0.19 |
| KEGG_PENTOSE_PHOSPHATE_PATHWAY | 25 | 0.47 | 0.07 | 0.18 |
| KEGG_STARCH_AND_SUCROSE_METABOLISM | 39 | 0.44 | 0.06 | 0.18 |
| KEGG_MTOR_SIGNALING_PATHWAY | 47 | 0.40 | 0.04 | 0.17 |
| KEGG_GLYCOSPHINGOLIPID_BIOSYNTHESIS_LACTO_AND_NEOLACTO_SERIES | 25 | 0.46 | 0.08 | 0.21 |
| KEGG_NUCLEOTIDE_EXCISION_REPAIR | 44 | 0.45 | 0.10 | 0.21 |
| KEGG_O_GLYCAN_BIOSYNTHESIS | 21 | 0.46 | 0.08 | 0.23 |
| KEGG_HEDGEHOG_SIGNALING_PATHWAY | 54 | 0.36 | 0.06 | 0.22 |
| KEGG_GLYOXYLATE_AND_DICARBOXYLATE_METABOLISM | 16 | 0.51 | 0.13 | 0.24 |
| KEGG_PROSTATE_CANCER | 84 | 0.33 | 0.05 | 0.24 |
| KEGG_CITRATE_CYCLE_TCA_CYCLE | 29 | 0.47 | 0.15 | 0.23 |
| KEGG_REGULATION_OF_ACTIN_CYTOSKELETON | 197 | 0.32 | 0.07 | 0.24 |
| KEGG_SELENOAMINO_ACID_METABOLISM | 20 | 0.46 | 0.12 | 0.23 |
| KEGG_ALANINE_ASPARTATE_AND_GLUTAMATE_METABOLISM | 28 | 0.41 | 0.10 | 0.24 |
| KEGG_ENDOMETRIAL_CANCER | 51 | 0.36 | 0.08 | 0.23 |
| KEGG_GAP_JUNCTION | 82 | 0.33 | 0.07 | 0.24 |
| KEGG_BASAL_TRANSCRIPTION_FACTORS | 31 | 0.42 | 0.13 | 0.23 |
| KEGG_WNT_SIGNALING_PATHWAY | 145 | 0.30 | 0.06 | 0.24 |
| KEGG_AXON_GUIDANCE | 124 | 0.31 | 0.08 | 0.24 |
| KEGG_INOSITOL_PHOSPHATE_METABOLISM | 53 | 0.33 | 0.08 | 0.24 |
| KEGG_ENDOCYTOSIS | 158 | 0.28 | 0.07 | 0.25 |
|  |  |  |  |  |
| *Gene sets enriched in phenotype low risk score* |  |  |  |  |
| KEGG_CYTOKINE_CYTOKINE_RECEPTOR_INTERACTION | 236 | -0.59 | 0.00 | 0.00 |
| KEGG_COMPLEMENT_AND_COAGULATION_CASCADES | 62 | -0.64 | 0.00 | 0.00 |
| KEGG_HEMATOPOIETIC_CELL_LINEAGE | 73 | -0.68 | 0.00 | 0.00 |
| KEGG_DRUG_METABOLISM_CYTOCHROME_P450 | 53 | -0.59 | 0.00 | 0.00 |
| KEGG_ARACHIDONIC_ACID_METABOLISM | 51 | -0.53 | 0.00 | 0.01 |
| KEGG_CHEMOKINE_SIGNALING_PATHWAY | 175 | -0.51 | 0.00 | 0.01 |
| KEGG_SYSTEMIC_LUPUS_ERYTHEMATOSUS | 40 | -0.66 | 0.00 | 0.01 |
| KEGG_PRIMARY_IMMUNODEFICIENCY | 32 | -0.79 | 0.00 | 0.01 |
| KEGG_INTESTINAL_IMMUNE_NETWORK_FOR_IGA_PRODUCTION | 33 | -0.69 | 0.01 | 0.01 |
| KEGG_JAK_STAT_SIGNALING_PATHWAY | 140 | -0.48 | 0.00 | 0.01 |
| KEGG_ASTHMA | 16 | -0.71 | 0.00 | 0.01 |
| KEGG_NATURAL_KILLER_CELL_MEDIATED_CYTOTOXICITY | 113 | -0.54 | 0.01 | 0.01 |
| KEGG_CELL_ADHESION_MOLECULES_CAMS | 105 | -0.53 | 0.00 | 0.01 |
| KEGG_TYPE_I_DIABETES_MELLITUS | 22 | -0.67 | 0.00 | 0.02 |
| KEGG_LEISHMANIA_INFECTION | 50 | -0.59 | 0.01 | 0.02 |
| KEGG_CYTOSOLIC_DNA_SENSING_PATHWAY | 47 | -0.52 | 0.01 | 0.03 |
| KEGG_GRAFT_VERSUS_HOST_DISEASE | 19 | -0.79 | 0.00 | 0.02 |
| KEGG_NEUROACTIVE_LIGAND_RECEPTOR_INTERACTION | 247 | -0.38 | 0.00 | 0.02 |
| KEGG_METABOLISM_OF_XENOBIOTICS_BY_CYTOCHROME_P450 | 55 | -0.51 | 0.01 | 0.02 |
| KEGG_ALLOGRAFT_REJECTION | 17 | -0.75 | 0.02 | 0.03 |
| KEGG_LEUKOCYTE_TRANSENDOTHELIAL_MIGRATION | 105 | -0.46 | 0.01 | 0.04 |
| KEGG_HISTIDINE_METABOLISM | 23 | -0.56 | 0.01 | 0.04 |
| KEGG_FATTY_ACID_METABOLISM | 37 | -0.53 | 0.02 | 0.04 |
| KEGG_TOLL_LIKE_RECEPTOR_SIGNALING_PATHWAY | 91 | -0.46 | 0.01 | 0.05 |
| KEGG_BETA_ALANINE_METABOLISM | 19 | -0.56 | 0.01 | 0.05 |
| KEGG_AUTOIMMUNE_THYROID_DISEASE | 30 | -0.55 | 0.02 | 0.06 |
| KEGG_PRION_DISEASES | 34 | -0.49 | 0.03 | 0.07 |
| KEGG_NOD_LIKE_RECEPTOR_SIGNALING_PATHWAY | 53 | -0.49 | 0.04 | 0.08 |
| KEGG_ETHER_LIPID_METABOLISM | 25 | -0.47 | 0.01 | 0.08 |
| KEGG_GLYCOSAMINOGLYCAN_DEGRADATION | 20 | -0.54 | 0.04 | 0.08 |
| KEGG_MAPK_SIGNALING_PATHWAY | 249 | -0.33 | 0.00 | 0.09 |
| KEGG_VALINE_LEUCINE_AND_ISOLEUCINE_DEGRADATION | 38 | -0.53 | 0.07 | 0.09 |
| KEGG_RETINOL_METABOLISM | 47 | -0.44 | 0.03 | 0.09 |
| KEGG_PROPANOATE_METABOLISM | 28 | -0.54 | 0.06 | 0.09 |
| KEGG_B_CELL_RECEPTOR_SIGNALING_PATHWAY | 67 | -0.47 | 0.07 | 0.09 |
| KEGG_FC_EPSILON_RI_SIGNALING_PATHWAY | 74 | -0.40 | 0.02 | 0.09 |
| KEGG_ANTIGEN_PROCESSING_AND_PRESENTATION | 53 | -0.47 | 0.06 | 0.09 |
| KEGG_T_CELL_RECEPTOR_SIGNALING_PATHWAY | 98 | -0.44 | 0.08 | 0.09 |
| KEGG_TRYPTOPHAN_METABOLISM | 30 | -0.46 | 0.04 | 0.09 |
| KEGG_CALCIUM_SIGNALING_PATHWAY | 166 | -0.33 | 0.01 | 0.10 |
| KEGG_DILATED_CARDIOMYOPATHY | 85 | -0.38 | 0.03 | 0.10 |
| KEGG_ABC_TRANSPORTERS | 44 | -0.40 | 0.03 | 0.12 |
| KEGG_APOPTOSIS | 77 | -0.38 | 0.07 | 0.12 |
| KEGG_HYPERTROPHIC_CARDIOMYOPATHY_HCM | 79 | -0.38 | 0.04 | 0.12 |
| KEGG_VIRAL_MYOCARDITIS | 48 | -0.43 | 0.07 | 0.12 |
| KEGG_ALDOSTERONE_REGULATED_SODIUM_REABSORPTION | 39 | -0.40 | 0.04 | 0.11 |
| KEGG_BUTANOATE_METABOLISM | 30 | -0.47 | 0.06 | 0.12 |
| KEGG_PEROXISOME | 74 | -0.40 | 0.08 | 0.12 |
| KEGG_VASCULAR_SMOOTH_MUSCLE_CONTRACTION | 108 | -0.33 | 0.04 | 0.13 |
| KEGG_OTHER_GLYCAN_DEGRADATION | 15 | -0.50 | 0.12 | 0.14 |
| KEGG_GLYCEROLIPID_METABOLISM | 38 | -0.38 | 0.07 | 0.14 |
| KEGG_LYSOSOME | 107 | -0.37 | 0.11 | 0.14 |
| KEGG_PPAR_SIGNALING_PATHWAY | 63 | -0.37 | 0.10 | 0.16 |
| KEGG_COLORECTAL_CANCER | 61 | -0.34 | 0.09 | 0.19 |
| KEGG_NICOTINATE_AND_NICOTINAMIDE_METABOLISM | 21 | -0.41 | 0.14 | 0.20 |
| KEGG_AMYOTROPHIC_LATERAL_SCLEROSIS_ALS | 48 | -0.34 | 0.11 | 0.20 |
| KEGG_ADIPOCYTOKINE_SIGNALING_PATHWAY | 62 | -0.32 | 0.10 | 0.23 |
| KEGG_SMALL_CELL_LUNG_CANCER | 77 | -0.33 | 0.13 | 0.22 |
| KEGG_PROXIMAL_TUBULE_BICARBONATE_RECLAMATION | 23 | -0.40 | 0.17 | 0.24 |
| KEGG_ACUTE_MYELOID_LEUKEMIA | 54 | -0.34 | 0.17 | 0.24 |
| KEGG_RIBOSOME | 86 | -0.49 | 0.29 | 0.24 |
| KEGG_PHOSPHATIDYLINOSITOL_SIGNALING_SYSTEM | 75 | -0.31 | 0.15 | 0.25 |
| KEGG_SNARE_INTERACTIONS_IN_VESICULAR_TRANSPORT | 38 | -0.34 | 0.18 | 0.25 |
